# Indirect mobilisation and violence legitimation through influencers on alternative platforms

**DOI:** 10.1111/bjso.70079

**Published:** 2026-04-03

**Authors:** Darja Wischerath, Olivia Brown, Lukasz Piwek, Brittany I. Davidson

**Affiliations:** ^1^ School of Management University of Bath Bath UK

**Keywords:** collective action, conspiracy theories, indirect mobilisation, online influencers, self‐categorisation theory, violence legitimation

## Abstract

Existing mobilisation literature has largely focused on groups and collective sensemaking processes as the primary drivers of collective action. However, online influencers have emerged as key leaders and mobilisers, which can shape collective action through one‐to‐many communication. Using self‐categorisation theory, we examine indirect mobilisation and the legitimation of violence during the August 2024 UK riots through a case study of a far‐right influencer's Telegram channel. The dataset consists of 230 posts and 156 pieces of multimedia content from the Tommy Robinson News Telegram channel from 29th July to 7th August 2024. We employed an abductive thematic analysis approach, revealing how throughout the progression of the riots, posts in the Telegram channel construct group identities, establish epistemic authority and leadership legitimacy, and legitimise violence. Our findings extend the social identity approach of mobilisation into the digital realm, revealing how broadcast‐style, unidirectional affordances of Telegram channels can impact the dynamics of leadership, identity construction and mobilisation of (violent) collective action.

## INTRODUCTION

In this article, we examine indirect mobilisation and the legitimation of violence during the August 2024 UK riots through a case study of far‐right influencer Tommy Robinson's Telegram channel. Existing mobilisation literature has largely focused on groups and collective sensemaking processes as the primary drivers of collective action (Brown, Lowery, et al., 2022; Smith et al., 2015). However, the shifting landscape of online social media platforms has introduced affordances (such as algorithmic amplification, closed one‐to‐many communications and monetisation) that amplify some voices far beyond others (Brown, Smith, et al., 2022). This may affect mobilisation efforts, as group consensus may no longer be the driving factor behind popular narratives. Examining leadership and mobilisation is therefore crucial to understanding how action is mobilised and legitimised in online spaces. While previous literature around leadership and mobilisation has focused on offline settings and elected leaders such as politicians (Ntontis et al., 2024; Reicher & Hopkins, 1996), we focus on online influencers and thought‐leaders with large social media followings. Utilising data from far‐right influencer Tommy Robinson's Telegram channel during the 2024 UK riots, we examine how online influencers can indirectly mobilise crowds through discursive framing of events and setting norms, through a self‐categorisation theory lens (Reicher & Hopkins, 1996).

### Mobilisation in an online age

Online spaces play a crucial role in organising protests and mobilising attendance across a diverse set of political movements and contexts. Social media platforms have been crucial for disseminating information during the Black Lives Matter protests (Brown, Lowery, et al., 2022), and Discord group chats were used to organise the far‐right Charlottesville Rally (Blout & Burkart, 2023), enabling real‐time coordination and documentation of events.

Furthermore, previous research has demonstrated that platform affordances affect offline mobilisation: Smith et al. (2023) demonstrated that protest attendance was positively associated with previous likes on relevant posts during an anti‐Brexit march. Mobilisation literature has to date primarily focused on the group as a site of mobilisation (e.g. Brown, Lowery, et al., 2022); however, recent work has begun to examine the role of online opinion leaders in shaping narratives and identities to mobilise audiences (Maxwell et al., 2025). This development is crucial as there is a tension between current models of mobilisation and how information is spread and encountered online, as well as the affordances of online platforms. In recent years, affordances shifted away from equal amplification of voices, for example, through chronological timeline feeds, and towards algorithmic amplification of popular content and creators (Brown, Smith, et al., 2022; Schellingerhout et al., 2025; Whittaker et al., 2021).

Additionally, increased restrictions on commenting and the rise of broadcast‐style communication, such as Telegram and Instagram broadcast channels, where content is delivered to a dedicated, unified feed with limited or no ability for followers to respond, have become more popular, further restricting dialogue within communities. Algorithmic systems compound this effect by propelling certain communications from key figures and their followers to the top of timelines, creating a perception of consensus and established norms even when broader community agreement may not exist (Brady et al., 2023; Metzler & Garcia, 2024). These affordances create online spaces where leaders can dominate discourse with minimal input from the wider group, instead creating content designed to be shared and spread within and across platforms, thus shifting the way that groups engage in dialogue and create consensus around beliefs, norms and actions (Metzler & Garcia, 2024). Previous mobilisation research has primarily examined speeches given by politicians (Haslam et al., 2023; Ntontis et al., 2024; Rapley, 1998; Reicher & Hopkins, 1996); however, short‐form and multimedia communications are increasingly used by group leaders to communicate with their followers (e.g. Maxwell et al., 2025). In addition to prolific users emerging as group leaders (Maxwell et al., 2025), political influencers strategically use communications to spread ideology and mobilise their followers (Rothut et al., 2024).

Rothut et al. (2024) conceptualise far‐right influencers as “individual actors who operate under their real name or an alias, position themselves publicly through political content, and advocate far‐right ideology via social media. They can serve as parasocial opinion leaders for their followers and use this mechanism to persuade and mobilise” (p. 7124). Online spaces, from mainstream to alternative, unmoderated platforms, are an important space for far‐right actors to disseminate ideology, recruit new followers and maintain communities (Baele et al., 2023; Lewis, 2018; Rothut et al., 2024). Spanning a wide, connected web, far‐right actors maintain a presence across mainstream and alternative platforms, from YouTube (Lewis, 2018) to Parler (Baele et al., 2023) and Telegram (Urman & Katz, 2022).

Expanding on previous work on leadership in mobilisation and online movement communication (Brown, Lowery, et al., 2022; Haslam et al., 2023; Maxwell et al., 2025; Ntontis et al., 2024; Reicher & Hopkins, 1996), this article examines mobilisation processes during the August 2024 UK riots following the Southport attack through a case study of Tommy Robinson's Telegram channel. Here, we focus on the communications of one individual who acts as an online political influencer, that is, intentionally positioning himself and his speech as a thought leader for his followers (Lewis, 2018) in the setting of a public Telegram channel solely aimed at communicating with his followers, allowing us to examine how one leader's communications can set norms, values and boundaries that facilitate collective action and legitimise violence. We adopted the term “parasocial opinion leader” to examine Tommy Robinson's communications on Telegram, drawing on Stehr et al. (2015)'s definition of parasocial opinion leadership as media personalities who influence their audiences' opinions and attitudes through parasocial relationships by providing information, orientation or arousing interest in topics.

### Online leadership and mobilisation

The decentralised nature of online communications has facilitated a shift in the dynamics of leadership beyond traditional elected group leaders. Political influencers draw on large networked audiences and platform affordances to spread ideology and mobilise their audience (Riedl et al., 2023; Rothut et al., 2024). Through repeated exposure to an influencer's content, audiences can develop a one‐sided parasocial relationship with them, feeling a perceived sense of intimacy and closeness despite the absence of reciprocal social interactions (Horton & Wohl, 1956; Stehr et al., 2015). Parasocial opinion leadership is important as information is more trusted when it comes from sources with shared social bonds (Rothut et al., 2024). Parasocial opinion leadership was proposed by Stehr et al. (2015) as a phenomenon that “is based on viewers' perceptions and emerges if (a) a media user ascribes certain attributes to a media communicator based on a parasocial relationship, which (b) allows for a gradual influence of the media personality on the user's opinions and attitudes by fulfilling at least one of three functions: information and reduction of complexity, orientation, or arousal of interest.” Influencers who (intentionally) cultivate these parasocial bonds thus occupy a uniquely powerful position in the information ecosystem, able to shape their followers' attitudes and steer collective actions differently from how parties and mainstream media outlets do (Stehr et al., 2015). Crucially, influencers make active, strategic decisions about how they position themselves as leaders and communicate with their audiences (Benford & Snow, 2000; Haslam et al., 2020).

In the context of mobilisation of collective action, leaders make strategic decisions about whether to directly mobilise followers through explicit calls for action or indirectly through collective action frames and interpretations of events. Indirect mobilisation involves leaders setting collective action frames that identify problems, attribute blame and motivate action, as well as broader attitudes around what to believe about the group and its objectives (Benford & Snow, 2000; Bestvater & Loyle, 2025; Jost & Dogruel, 2023). For example, Haslam et al. (2023) demonstrate how Trump's influence over his supporters during the January 6th Capitol attack operated through framing general goals (“stop the steal”) without providing specific instructions, leaving followers to creatively interpret and enact these directives while maintaining plausible deniability. In its most extreme form, Amman and Meloy (2024) term this phenomenon stochastic terrorism: the increase in probability of violence without direct commands, operating through inflammatory rhetoric that creates conditions where violence becomes statistically probable but remains unpredictable on an individual level. Unlike legal incitement, which requires demonstrable intent to cause imminent harm, stochastic terrorism occurs through diffusion of responsibility, specifically the use of conspiracy narratives and dehumanising rhetoric to create climates of outrage and existential threat (Amman & Meloy, 2024; Angove, 2024).

Indirect mobilisation strategies, especially when paired with evolving platform affordances, allow leaders to respond to real‐world events and engage with audiences in unprecedented ways. For example, Trump was posting actively during the storming of the Capitol on January 6, 2021 (BBC, 2021). Furthermore, unlike speeches that are largely delivered in‐person (Ntontis et al., 2024) or transmitted via TV (Reicher & Hopkins, 1996), online audiences tend to be more dispersed both geographically and temporally. Indirect mobilisation strategies thus allow leaders to include a larger ingroup in their communications, setting group norms beyond the immediate event. This approach is particularly useful when trying to understand how protests spread from localised events to nationwide violent unrest. Therefore, we extend previous literature (Bestvater & Loyle, 2025; Haslam et al., 2023; Jost & Dogruel, 2023) and examine how indirect mobilisation contributed toviolence and the spread of unrest during the August 2024 UK riots following the Southport attack.

### Social identity approach to mobilisation

The social identity approach to mobilisation proposes that social group membership informs our self‐concept, understanding of shared norms and values, and thus affects propensity to action (Portice & Reicher, 2018; Reicher & Hopkins, 1996; Turner et al., 1979). Specifically, self‐categorisation theory posits that the boundaries of an activated social identity determine the extent of collective action (i.e., who acts together), while the content of the group identity (i.e., its norms, values, and stereotypes) defines the type of action (Finlay, 2014; Herrera & Reicher, 1998; Reicher & Hopkins, 1996).

Crucially, these category definitions are not static but are continually discursively constructed. That is, group leaders, such as politicians or online influencers, rhetorically define the boundaries and content of social categories in a way that makes their position appear normative for the largest possible proportion of the group. Furthermore, leaders gain authority and support through presenting themselves as representative of the group, acting in its best interests and defending it against threats (Portice & Reicher, 2018). For example, Rapley (1998) illustrates how an Australian right‐wing populist politician constructs legitimacy for her positions and right to mobilise the group by presenting herself as an “ordinary Australian” and a prototypical member of this discursively constructed social category. This process often involves framing current events in moral terms, highlighting a threat to the ingroup. Indeed, moral outrage and moral convictions have been identified as key antecedents of collective action, motivating and uniting groups to act (van Zomeren, 2013).

Beyond mobilising collective action, how groups are defined also constructs norms and values around actions and beliefs, including whether violence is warranted towards the outgroup. As a non‐normative action, violence must first be legitimised within a social network in order to become an acceptable action for group members (Bandura, 2004; Kruglanski et al., 2022). The construction of threat from the outgroup towards the ingroup represents a key legitimating mechanism (Portice & Reicher, 2018). For example, the perception that the outgroup is engaging, or intending to engage, in unjust aggression against the ingroup can morally legitimate the use of violence in response (Bandura, 2004; Ferris et al., 2019). Furthermore, the construction of existential threat to the ingroup justifies the use of violence, as the group's existence is under attack (Portice & Reicher, 2018). Crucially, it is the communication of threat, rather than experience with it, that seems inherent to the success of mobilisation and justification of violence (Portice & Reicher, 2018).

In the present research, we draw on the above theoretical approaches to examine how an online influencer engages in identity entrepreneurship to indirectly mobilise his followers and legitimate violence through discursive framing rather than explicit instruction. Specifically, we examine how these processes unfold through the affordances of online broadcast‐style communication, which enables identity work that is both responsive to real‐time events and directed by a single influential voice.

### Case context: The august 2024 UK riots

The August 2024 riots across England followed a knife attack at a Taylor Swift‐themed dance class in Southport on 29th July, which killed three young girls and injured several others (Halliday, 2025). Misinformation rapidly spread online, falsely claiming the attacker was an asylum seeker or Muslim immigrant, prompting the police to confirm he was born in Cardiff to Rwandan parents and later release his name (Quinn, 2024b). The unrest occurred against a backdrop of earlier incidents in July, including riots in the Harehills area of Leeds involving Roma communities, which some commentators later cited as evidence of ‘two‐tier’ policing practices (M. Brown, 2024).

Prior to the Southport attack, Tommy Robinson organised the ‘Unite the Kingdom’ rally in London on 27th July, claiming to have drawn 100,000 supporters to Trafalgar Square. During this event, he was detained under the Terrorism Act and subsequently fled the country (Dixon & Eastham, 2024; Quinn, 2024a). Following the Southport incident, Robinson used his substantial digital presence on platforms including X (formerly Twitter) and Telegram to comment on events and criticise government responses. His communications during this period have since become the subject of legal proceedings, with Robinson currently facing harassment charges (*Tommy Robinson Pleads Not Guilty to Harassment of Journalists*, 2025).

### The current study

In the current study, we extend previous work on leadership and collective action (Jost & Dogruel, 2023; Ntontis et al., 2024; Reicher & Hopkins, 1996) by examining indirect mobilisation and violence legitimation on an alternative social media platform during the 2024 riots in the UK following the Southport attack. Specifically, we analyse Tommy Robinson's Telegram channel. Whilst Tommy Robinson was active and gathered more reach on X, we chose Telegram as most organisation of local protests happened on that platform, and his posts were most likely to reach active protesters there through shares (Institute for Strategic Dialogue, 2024).

Using a social identity approach lens, we therefore ask:

1. How are group categories constructed throughout the course of the riots?
2. How does Tommy Robinson legitimate himself as a leader?
3. What mobilisation strategies are used throughout the riots?
4. How is violence constructed as a legitimate course of action?

## MATERIALS AND METHODS

Ethical approval has been granted by the University of Bath (REF: 6959–8501).

### Data

This study focuses on Tommy Robinson's broadcasting channel on Telegram. Telegram is a messaging platform emphasising privacy and security, popular with extremist groups and users banned from mainstream social media platforms due to its limited moderation (Telegram, n.d.; Urman & Katz, 2022; Walther & McCoy, 2021). We chose Telegram due to its central role in the organisation of protests, often by far‐right and conservative groups (Jost & Dogruel, 2023; Urman & Katz, 2022). Furthermore, Telegram differs from the majority of social networking platforms as it does not possess an algorithmic homepage in which users are recommended content. Instead, it hosts group chats and broadcast‐style channels where channel hosts can send posts to subscribers. Telegram can therefore function as a space reserved for the ingroup with no external input or challenge from outgroup voices. Moreover, the broadcast format means that posts are designed to be consumed and shared rather than discussed. Telegram channels thus offer a glimpse into how opinion leaders construct narratives for their existing followers without interference from outgroup voices.

The dataset for this article is taken from Tommy Robinson's Telegram channel *Tommy Robinson News*, which is run by Robinson and an administrative team. At the time of data collection, the channel had over 90,000 subscribers and the comment function was turned off. We analysed 230 posts and 156 pieces of multimedia content (videos and photos) from Tommy Robinson's Telegram channel from 29th July 2024 to 7th August 2024 (henceforth posts), spanning the period from the initial Southport attack through the subsequent nationwide riots. This time period was selected to capture the evolution of mobilising discourse from the immediate aftermath of the Southport attack through the peak and subsequent dissolution of nationwide riots. Video, photo and text data, as well as metadata such as emoji reactions (Appendix A shows the most commonly used emojis in the Telegram channel), were collected through the Telethon API (Lonami, 2016), as well as the download function on Telegram Desktop. The latter was used to enable more immersive qualitative coding in the same format as the posts would be displayed to readers.

**FIGURE 1:**
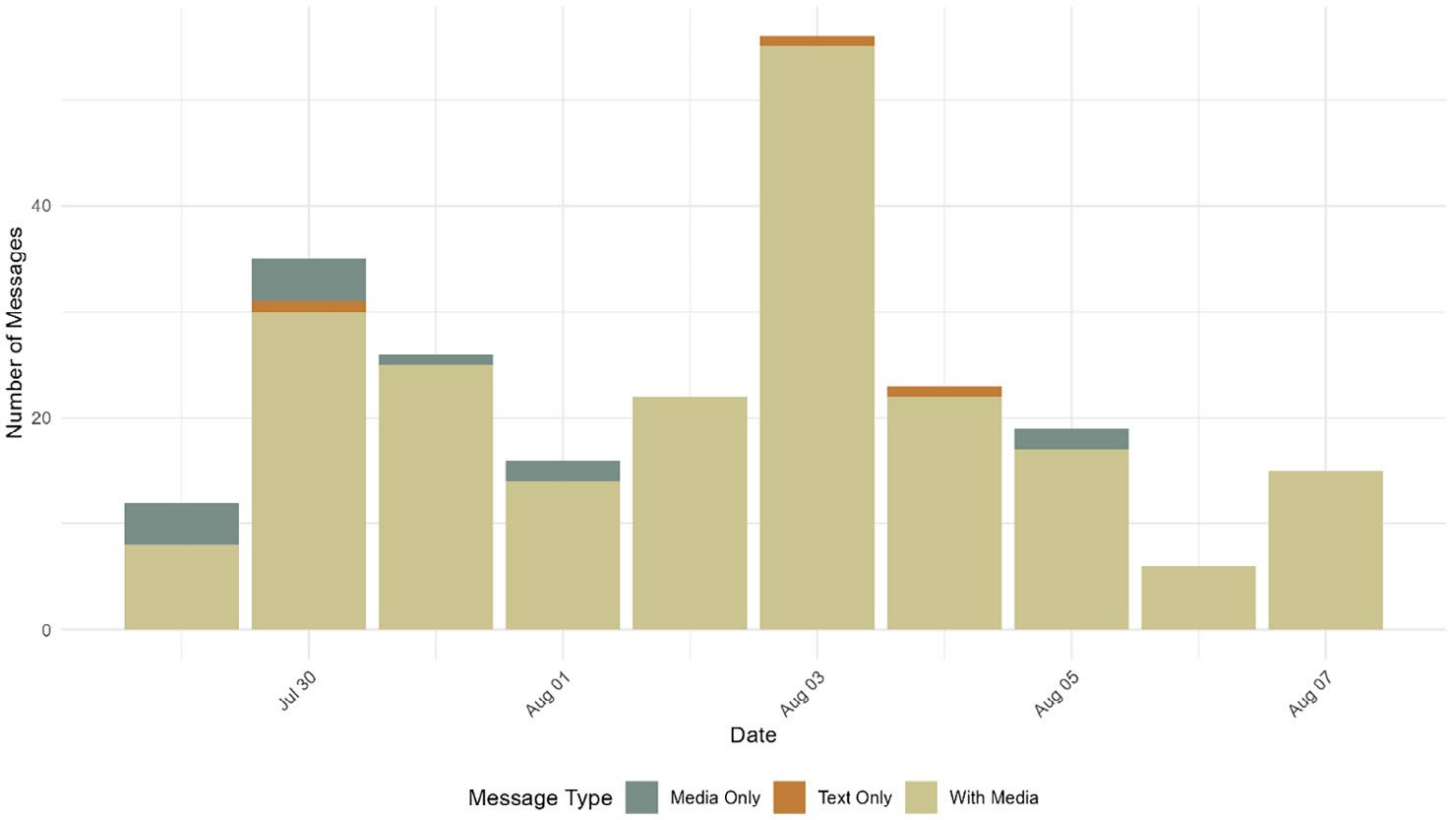
Bar plot showing number of posts that are media only, text only or text accompanied by media per day.

Most posts contained multimedia elements, such as videos or photos, in addition to text (see Figure 1). Videos included footage of the Unite the Kingdom rally (mean length 05:29 min), protest footage (mean length 00:27 s), clips from mainstream media reports (mean length 02:43 min), reports from Urban Scoop, Robinson's media company (mean length 01:58 min) and direct video messages from Robinson (mean length 06:14 min), with video lengths ranging from 00:04 s to 14:03 min.

### Analytic approach

Our analytical strategy was informed by the social identity approach to mobilisation, specifically self‐categorisation theory (Turner et al., 1987; Reicher & Hopkins, 1996), and employed an abductive thematic analysis approach, combining inductive coding with theoretical interpretation (Braun & Clarke, 2006). This approach enabled us to identify patterns in the data whilst connecting emergent themes to established theoretical frameworks around mobilisation and violence legitimation (see also Ntontis et al., 2024).

The analysis proceeded through six phases following Braun and Clarke's (2006) framework. The lead author first familiarised themselves with the dataset through repeated reading of all posts and viewing of all multimedia content. The first author coded all posts inductively using MAXQDA software (VERBI Software, 2023), generating descriptive codes that captured manifest content and latent meanings. Multimedia content was analysed holistically with accompanying text, with particular attention paid to how visual elements reinforced or extended text. Videos and photos were watched and analysed together with their accompanying text if available and were coded for their framing and contextual contribution to the overall post message. This holistic analysis approach was chosen to mimic the affordances of how text is embedded in images and is viewed on Telegram and other social media platforms (see also Brown, Lowery, et al., 2022). This is particularly important for our dataset, where most posts were accompanied by a photo or video.

Videos featuring Tommy Robinson speaking directly to the camera received more detailed analysis given our focus on his role as a mobilising leader, with these communications transcribed using MAXQDA's transcription feature and coded as primary textual data. Initial codes were then collated into potential themes, which were reviewed by all co‐authors. In the final phase, themes were refined and defined through explicit connection to self‐categorisation theory, examining how posts constructed ingroup and outgroup boundaries, legitimated violence and mobilised collective action.

## RESULTS

Our analysis reveals how the Tommy Robinson News Telegram channel constructs group identities, establishes epistemic authority and legitimacy as an opinion leader and mobilises collective action. These processes unfolded through four interconnected themes: Ingroup category construction, leadership construction, connection of outgroups and violence legitimation (see Table 1). Throughout the dataset, populist rhetoric was continually used, positioning “the people” against the government and a constructed dangerous other. Notably, all themes could be identified within the majority of posts. Posts that primarily focused on positioning the ingroup would, for example, simultaneously discuss the government and use causal, conspiratorial narratives to describe events depicted in accompanying videos or photos. Therefore, while we aimed to curate and present the most relevant excerpts, each quote necessarily contains multiple themes and mechanisms.

**TABLE 1:** Themes and subthemes.

Theme	Subthemes	Description
Flexible boundaries of ingroup identity	Ingroup as “the people”	Posts describing protesters as “the people” and creating an opinion majority status
	Boundary flexibility	Category boundaries are narrowed or expanded to create the greatest differences between groups
	Group values	Posts emphasising ingroup values around child safety and national pride
	Positive movement identity	Posts highlighting the success of the Unite the Kingdom rally
Establishing opinion leadership	Establishing prototypicality	The leader establishes prototypicality through shared values with the group and by posing their own experiences as the future of the group
	Prescient knowledge	The leader acts as a prophet and an unheard victim
	Articulating future vision	Current events are part of a larger existential struggle
Connecting outgroups through conspiracy narratives	Muslims and immigrants: The dangerous other	Muslims are framed as a dangerous violent other that threatens the existence of the ingroup
	Government, media and police: Elite authorities	The system is intentionally abandoning the people; the police are failed protectors
	Conspiracy theories	Outgroups are connected through explicit and implicit conspiracy theories
Indirect Mobilisation and violence legitimation	Anger and child protection as moral justifications	Cause of anger morally justifies violent outbursts
	Framing violence as an inevitable last resort	Violence is encouraged by the government as a means of communication
	The real violence is happening to the ingroup	Examples of violence against the ingroup
	Condemning violence	Explicit condemnation of violence and appeals for calm

### The concerned British public: Flexible boundaries of Ingroup identity

Throughout the dataset, the ingroup category is consistently framed as “the people”: For example, posts describe initial protests as “*the people revolting after the government ignored the concerns of the public and wrote them all of as ‘far right’*.” This draws on populist rhetoric, emphasising the perceived opinion majority status of the ingroup while rejecting labels used by the government and mainstream media.

The boundaries of the ingroup undergo strategic shifts as events unfold in real life, with category boundaries shifting to emphasise conflict between the ingroup and outgroup. Initially focused on localised grief (“*the people of Southport*”), the ingroup expands as protests spread nationally, with protesters reframed as “*the concerned British public*” to suggest widespread sentiment and add a nationalist sentiment. Similarly, ingroup boundaries are strategically narrowed in posts featuring specific outgroups. For example, when left‐wing counter‐protesters appear, class‐based categories are emphasised: “*The communists are out in Manchester against the working class who have genuine concerns about their country and future for their children and grandchildren*”. Whereas racial categories are made salient during clashes with Muslim counter protesters: “*Muslim mobs running through Sheffield city centre just attacking white people at random*.”

The flexibility of ingroup boundaries can serve to highlight long‐standing group conflict or emphasise existential threat to the ingroup. For example, emphasising working‐class identity taps into the longstanding narratives surrounding British authentic nationhood, simultaneously evoking racial imagery of whiteness. This is frequently seen in populist communications that capitalise on the racialisation of the working class as white and ‘left behind’, positioning them as representative of the ‘people’ or demos (Mondon & Winter, 2020). Similarly, racial categorisation draws upon white replacement fears, positioning any harm against white individuals as an attack on the broader social order and redefining who belongs to the public. The intersection of these identities becomes explicit when alternative media is praised for “*giving the white working class a voice*”, highlighting the perceived loss of representation and autonomy in mainstream media and government (Feola, 2021).

Despite flexible boundaries, the ingroup's core values remain stable and are constantly mentioned: child safety and national pride. These stable values enable the creation of superordinate identities that transcend traditional divisions. As riots escalate beyond England, posts celebrate “*Catholics and Protestants united in Belfast for the future of their children*”, highlighting how traditional divides are transcended to protect children from the ultimate enemy. Similarly, international solidarity is highlighted: “*French nationalists show their support for their European neighbours in the UK by highlighting the horrific murders of 3 little girls in Southport [Union Jack and French flag emojis]*”.

The consistent return to child safety serves a dual purpose of tying genuine emotions surrounding the Southport attack to larger grievances and concerns around child protection. Notably, child safety is both a common populist rhetorical device and a coded reference for right‐wing extremist narratives about white replacement fears (Essex, 2024).

As we explore further below, the ingroup is presented as under attack from multiple groups and characterised as “*forgotten, abused, neglected*”. Despite the continuous victimisation narrative, early posts feature positive sentiments about the movement through recaps of the *Unite The Kingdom* rally, organised by Tommy Robinson, which occurred days prior to the attack:

We came, we saw, we conquered. On Saturday, 100,000 patriots marched through the streets of London, and filled Trafalgar Square in scenes never witnessed before. We're building a resistance that clearly terrifies the establishment [union jack emoji] The future belongs to us!

These posts emphasise the success of the movement, including frequent references to the size of the event. The ingroup is positioned as a threat to the elites and evokes imagery of war and revolution. Other posts feature videos of key speakers during the event who talk positively about the change the ingroup is about to bring to society: “*there is no political solution…you…the people, are the solution*.” This functions as a positive mobilising force, reminding readers of the greater goal of the movement and emphasising individual roles within the movement's success, framing protest and action as a moral imperative.

### First they come for Tommy: Establishing opinion leadership

Tommy Robinson's role as an opinion leader is established through authenticity and shared identity markers. Prototypicality is key to establishing leadership, exemplified through several instances in the dataset:

For example, Tommy Robinson uses his status as a parent to position himself as a prototypical group member connected to the ingroup values and establish a connection with grieving communities, lending him additional epistemic authority: “*I remember taking my daughter to these sorts of dance classes [where the attack happened]. The joy in those little girls faces, the innocence of those children*.” By emphasising his personal connection, he underscores that this is an issue that could affect any parent in the group, thus reinforcing his status as a prototypical group member.

Similarly, his arrest at the Unite the Kingdom rally becomes evidence of prototypical status. Tommy Robinson is targeted by the government and police precisely because he represents the group so effectively: “*This is why the establishment are coming after me. I held the biggest patriotic rally this country has ever seen… First they come for Tommy*…” Here, his prosecution serves as an omen for the wider ingroup's future prosecution. Indeed, posts note how Prime Minister Keir Starmer “*labels everyone upset about the murder of 3 little girls by Axel Muganwa Rudakubana as ‘thugs’ and gives police more powers*”, prophesying the threat of arrest.

This prescient knowledge extends to violent escalations at protests: *As disorder spreads to Hartlepool, don't say I didn't warn you*. Here, Tommy Robinson positions himself as both prophet and unheard victim; if the government had listened to him, violence would never have happened. This both condemns violence and frames it as a last resort action, placing blame on the government for escalating the situation. Notably, Tommy Robinson also articulates a vision for the future beyond immediate events. Through video messages, often spanning five minutes or more, he encourages his audience to consider the wider implications of the riots:

You're gonna ask: Was it a mob of coked up drunk Englishmen, or was it a resistance of dedicated, fit, healthy, ready, British resistance, ‘cause that's what's needed. Lads you need to get in the gym. I almost sound like I'm preaching, but we need to be ready for what's coming. […] We either win this battle where we don't become minorities and don't see every time we sit in this country taken over by hostile jihadists, rapists, criminals. That's one outcome, or we win through peaceful resistance. We cause a revolution in this country, and the public are ready for it. Everyone's ready for it. The time is now

Very explicitly, the threat of extinction is brought up through Great Replacement conspiracy theory, conjuring images of war and battle, thus once again justifying the use of violence to defend the ingroup and one's very existence.

### Government puppets: Connecting outgroups through conspiracy narratives

As protests progress to riots, sophisticated conspiracy narratives are created to explain the multitude of perceived threats. Multiple outgroups – Muslims, immigrants, government, mainstream media and police – are connected through strategic juxtaposition of posts and explicit coordination claims.

Muslims and immigrants represent the “dangerous other”—a populist rhetorical device that increases existential threat perceptions by combining immediate violence with cultural replacement fears. The two categories are used almost interchangeably, that is, every immigrant is seen as Muslim, and all Muslims are perceived as immigrants. Posts emphasizse the incompatibility of this group with British values. Indeed, posts stereotype all Muslims and immigrants as complicit in violence, claiming that when “*a muslim lad went to his mosque and said he wants to kill non‐Muslims. Do you know what they did? They didn't report him. They prayed for him that morning. He then left the mosque in London and he went on a stabbing spree while screaming Allah Akbar!*” Furthermore, violence from this group is seen as a targeted existential threat to the ingroup. In a video transcript, Tommy Robinson highlights that attacks from Muslims target “*vulnerability and something […] sacred*” to the ingroup, such as their children, intensifying existential threat perceptions. This elicits moral disgust towards the outgroup, framing them as fundamentally threatening to the ingroup's values and existence, thus implicitly legitimising harm against them to protect the ingroup (Matsumoto & Hwang, 2012).

Government, mainstream media and the police form a second group: the elite authorities. The government is positioned as the most agentic and responsible group, influencing mainstream media and police to further their agenda while abandoning the people. After Prime Minister Keir Starmer condemned the riots, group boundaries narrowed to blame Starmer personally, evoking feelings of abandonment and contempt: “*This is on keir starmer. Instead of listening to genuine concerns of the public. You wrote them all off as ‘far right thugs’. And the media danced to it, even resurrecting the decade gone ‘EDL’ and blaming them*.”

The police, who are supposed to be protectors of public safety, are reframed as enforcers of the government's will, abandoning the people even further: “*They'll protect what the public don't want, but not the public. Yet they'll face off with the public but not what the public don't want*.” This is positioned as finalising Britain's descent into chaos, thus removing existing norms and barriers around enacting violence.

Throughout the dataset, the outgroups are connected through three simultaneous conspiracy mechanisms operating at different levels of explicitness. At the implicit level, the strategic juxtaposition of unrelated events creates impressions of coordinated threat without explicit claims:

Masked Muslims carrying weapons in Stoke and Blackburn, police too scared to intervene. Simultaneously, the concerned British public out in Blackpool, police setting their dogs onto them.

At the explicit level, government orchestration becomes the central explanatory framework, alleging that the “government have their puppets in the media targeting anyone pointing out their failings” and that this has “put a target on [protesters] heads” and “encouraged” violence against protesters by police and Muslims.

The overarching framework is provided by the Great Replacement conspiracy theory, providing a master narrative that connects demographic change, cultural transformation and elite betrayal into a coherent existential threat (Obaidi et al., 2022). Primarily emerging in video messages, Tommy Robinson warns of the Labour government's alleged plans:

They're replacing the British population with hostile, violent, aggressive migrants who will continuously vote for them. […] All the young white girls getting raped and tortured were collateral damage because Labour will continue to gain power from that bloc Muslim vote, so they don't give a fuck. In all honesty, your children don't matter to them. They don't care, okay? They don't care. They have a greater plan that they're playing, and it's like a 30, 40, 50, 60 year plan in place. And that is to flood this nation. It's not a mistake. They are going to destroy our identity, our culture. They're going to break us all down.

Conspiracy narratives are particularly effective for mobilisation purposes because they frame current events not as isolated incidents but as part of a deliberate, long‐term assault on group survival itself (Bartlett & Miller, 2010). Therefore, by positioning demographic change as intentional genocide rather than natural social evolution, Tommy Robinson can transform ordinary political disagreements into life‐or‐death struggles, thereby justifying extraordinary responses including violence. Using graphic and dehumanising language around the outgroups evokes disgust and moral outrage, reinforcing the perception of Muslims being incompatible with the ingroups’ existence and the government as the ultimate conspirator, willing to eradicate the ingroup for their own goals. Additionally, the conspiracy's temporal scope (“*a 30, 40, 50, 60 year plan*”) creates urgency whilst suggesting that conventional political processes are inadequate to address such systematic and patient destruction (see also Bartlett & Miller, 2010).

### Pushed too far: Indirect mobilisation and violence legitimation

Throughout the dataset, mobilisation efforts are largely indirect, relying on emotional appeals and providing targets against which outrage can be directed. The exception is posts about Tommy Robinson's arrest, encouraging readers to sign an online petition against Hope Not Hate, which appear throughout the dataset. However, one of the first posts about the Southport incident states: “*What will it take for you to be angry enough to do something about this?*”, creating a sense of urgency and fatalism about the attacks. The situation is portrayed as escalating further, and the readers' anger is required to change it.

Violence legitimation proceeds through three key mechanisms that progress and build on each other throughout the data. They serve to make violence appear consistent with ingroup values, rather than deviant, without explicitly condoning violence.

The deaths of three girls in Southport are frequently referred to in almost every post, particularly at the beginning of the dataset. Similarly, anger is a frequently mentioned emotion, inflamed by frequent rhetoric framing “the people” as angry or concerned, signalling danger about the future of the ingroup and highlighting existential threats:

Before I posted this. I asked myself “am I too angry?”. No I'm not, children are being murdered, our daughters are being murdered. None of us are feeling safe in our own country, in our own towns.

Similarly, harm to children is framed as a last straw in an already threatening world:

The British have been pushed too far. Once you start f*cking with their children, taking away their safety. What do you expect to happen?

An unidentified “you” intentionally takes away the safety of the children, violence becomes the only effective and way of saving the ingroup.

Furthermore, referring to previous riots in Leeds, violence is framed as the only way to get the government's attention:

As disorder spreads to Hartlepool, don't say I didn't warn you. They sent out the message that riots works over the last few weeks. Roma gypsies rioted, you handed them the kids back. Muslims rioted, yous dropped the charges. The government and “authorities” created this.

Here, violence is portrayed as a last resort, reluctantly adopted by the ingroup, justified by outgroups having successfully achieved their aims through rioting and received government concessions as a result. Violence is thus framed as antithetical to the ingroups values but must be taken as it is a behaviour norm set by the outgroup.

As violence escalates, posts emphasise the violence experienced by the ingroup, both from the police and from Muslims and other counter‐protesters. Violence from police against protesters is seen as the ultimate failing of the institution, highlighted by the arson of the Sunderland police station. This is again framed as an inevitable, yet necessary step to get the attention of the government:

Sunderland's previous main police station destroyed before a newer one is burned out. Instead of listening to genuine concerns of the public, @Keir_Starmer used the police as a weapon against them. This is on the government.

Similarly, describing protesters as far‐right is seen as an invitation to “emboldening” Muslims to enact violence against “the public” and “random white people”:

Two British men stabbed and attacked with hammers in Stoke by gangs of Muslims hyped up by @Keir_Starmer the media and far left lies. Instead of listening to their concerns, they branded the working class “far right thugs” with their sick rhetoric. Blood on their hands!

Throughout the dataset, Tommy Robinson employs strategic ambiguity and contradiction by explicitly condemning violence whilst simultaneously inflaming the emotions that fuel it. For example, in one video message, he begins with “*a total appeal for calm*” while reminding viewers that “*another man has come into their town with a balaclava and knife*”, acknowledging that “*the anger is boiled over*” and “*our country is in the gutter*”. Appeals to refrain from violence are accompanied by reminders about long‐term mobilisation, telling viewers that “*We ain't going win it [the country] back by throwing a few rocks at coppers*” and that “*[getting] years in prison […] [is] not helping the cause*”. Instead, viewers are commanded to “Galvanise the country. That's what we need to do. Unite people” and “prepare for what is to come”.

## DISCUSSION

The aim of this paper was to examine how online influencers indirectly mobilise collective action and justify violence as a legitimate means of action using a social identity approach lens. We examined this through a case study of Tommy Robinson's Telegram channel during the August 2024 UK riots following the Southport attack. Our findings extend existing indirect mobilisation and leadership literature (Haslam et al., 2023; Ntontis et al., 2024) by looking at online communications from political influencers. Addressing our four research questions, we find that group categories are constructed through flexible, shifting boundaries, expanding the ingroup from grieving locals to “the British people” whilst narrowing outgroup boundaries around the most salient threat at any given time (RQ1). Tommy Robinson's leadership is legitimised through identity entrepreneurship work that emphasises his prototypicality and epistemic authority, in part through establishing parasocial relationships (one‐sided relationships in which audiences develop a perceived sense of intimacy and closeness with a media figure; Stehr et al., 2015) with his audience (RQ2). Mobilisation efforts are predominantly indirect, including populist framing of groups, emotional appeals that elicit anger, contempt and disgust and the strategic use of conspiracy narratives that tie multiple outgroups into a coherent existential threat (RQ3). Violence is constructed as legitimate through these same mechanisms and is framed as an inevitable last resort due to government failures and threats to children, whilst explicit endorsement is carefully avoided (RQ4). Throughout, populist rhetoric pervades the messaging, reinforcing the divide between ‘the people’ and dangerous others and elites (Jost & Dogruel, 2023). Our findings extend existing indirect mobilisation and leadership literature (Haslam et al., 2023; Maxwell et al., 2025; Ntontis et al., 2024) by examining online communications from one political influencer during a time of unrest. Specifically, we demonstrate the identity entrepreneurship work that influencers engage in order to achieve group prototypicality and position themselves as parasocial opinion leaders, increasing the persuasiveness of their messaging and standing within the group. We also highlight how existing grievances are utilised to create conspiracy narratives and a cohesive narrative that ties multiple outgroups together, and how this helps to legitimise violence. In the following sections, we examine each contribution in turn.

### Parasocial leadership and indirect mobilisation online

Our findings extend the social identity approach of mobilisation into the digital realm, revealing how posts on an influencer's Telegram can alter the dynamics of identity construction and mobilisation through broadcast‐style, unidirectional communications. While previous research has predominantly examined SCT through analyses of political speeches delivered to physically present audiences (e.g. Ntontis et al., 2024; Portice & Reicher, 2018; Reicher & Hopkins, 1996), contemporary (political) communication now occurs primarily through digital channels. Additionally, the rise of political influencers highlights a new form of leadership through parasocial opinion leaders.

The shift to online communication introduces several affordances that transform how social categories are constructed. Unlike traditional speeches, which represent discrete communicative events, digital platforms like Telegram enable near‐continuous messaging through shorter‐format posts that create meaning over time through their collective impact. For example, in our analysis, 230 posts were sent over 10 days, with individual days ranging from six to 35 posts. Together, these posts formed a flexible narrative arc, with each message building upon the previous whilst responding to ongoing events. Our analysis demonstrated how category constructions shifted as events unfolded, which is a flexibility afforded by real‐time, continuous online communication rather than longer speeches. This responsiveness, combined with conspiracy narratives, allows influencers to adapt to contradictory or unexpected developments while retaining narrative coherence. Furthermore, indirect mobilisation tactics may be more sustainable, as too many direct calls to action can lead to message fatigue (Bestvater & Loyle, 2025). That is, setting norms and values through continued interpretation of current events can frame protest, and even violence, as a moral imperative, opening pathways of action for readers and encouraging them to act implicitly. Importantly, in the present case study, the indirect mobilisation tactics appeared to allow Tommy Robinson to mobilise followers while maintaining plausible deniability and potentially avoiding charges of legal incitement, though further research across different contexts and influencers is needed to generalise this pattern (Amman & Meloy, 2024; Angove, 2024). In the present case context, while Tommy Robinson was widely identified as a key figure during the unrests, he was not charged with incitement offences in relation to the riots at the time of writing (Gadher et al., 2024). However, when a complaint was made to the BBC Executive Complaints Unit regarding a presenter's questioning of whether Robinson's online content constituted incitement to hatred, the ECU ruled that the sharing of unsubstantiated claims about the attackers' background was sufficient to justify the line of questioning (BBC ECU, 2024).

This messaging may be particularly effective from online influencers through their nature as parasocial opinion leaders, as communications by those with whom we perceive ourselves to share social bonds are trusted more (Rothut et al., 2024). Our analysis highlights efforts within the channel to position Tommy Robinson as a prototypical, relatable group member, for example by highlighting his status as a parent and a prescient voice for the group. This prototypicality is strategically cultivated in order to maintain his status as group leader (Haslam et al., 2020; Reicher & Hopkins, 1996). Strong parasocial bonds between leaders and followers affect the persuasiveness of messages, as interpersonal communication has a stronger impact on attitudes and behaviour than mass communications (Rothut et al., 2024). Parasocial relationships can function as interpersonal bonds, meaning that norms, beliefs and interpretations of events shared by a parasocial opinion leader may be more readily accepted and acted upon by followers (Rothut et al., 2024; Stehr et al., 2015).

Self‐categorisation theory emphasises the role of interactions between leaders and followers to co‐construct identity and norms (Haslam et al., 2020; Hogg, 2001). However, we observed that the absence of dialogue affordances is a key feature of Telegram channels. In the *Tommy Robinson News* channel, the comment function was turned off, and Telegram does not notify channel owners of emoji reactions to posts. This means that the identity construction and mobilisation processes documented in our analysis operate in an essentially unidirectional manner: the influencer sets group norms, boundaries and values without direct input or challenge from followers. Engagement within the Telegram channel is constricted to sharing posts and emoji reactions, which we found were overwhelmingly affirming (Appendix A), further underscoring that the channel functions as an echo chamber rather than a site of discursive co‐construction. We argue that this absence of co‐construction does not constitute a limitation of our analysis but rather is a defining feature of certain communications of Telegram that facilitates the unilateral identity work observed here. Thus, we have explicated how group identity is constructed and how category boundaries and norms for action are established when driven by a single influential voice without negotiation.

### Conspiracies as unifying narratives

Previous research examining social identity processes in mobilisation has primarily focused on simple ingroup–outgroup dichotomies, typically involving a single salient outgroup (Turner et al., 1987; Reicher & Hopkins, 1996). Our findings reveal that online communications afford a more complex dynamic where multiple outgroups are present simultaneously, with their salience shifting in response to real‐world events. That is, rather than a single outgroup being present in the data, such as during Thatcher's and Kinnock's speeches during the miner's strikes (Reicher & Hopkins, 1996), our findings demonstrated the diversity of outgroups identified, dependent on how offline events were unfolding (e.g., Muslims, government officials, mainstream media and the police).

Notably, outgroups are often mentioned alongside each other and connected through conspiracy narratives. For example, violent altercations with the police are explained to be happening due to direct government instruction. Previous research has highlighted that perceived threats contribute to people turning to conspiracy narratives to process events and threats (Douglas et al., 2019). Conspiracy narratives thus serve as crucial mechanisms for managing complex, multifaceted experiences of the ingroup, providing overarching explanatory frameworks that can accommodate multiple outgroups within a coherent threat structure. Additionally, conspiracy narratives draw on existing shared cultural knowledge whilst tapping into broader ideological frameworks like Great Replacement theory (Obaidi et al., 2022). This means that connections of outgroups raise the perceived level of threat: the government is not merely allowing Muslim violence but planning to eradicate the entire ingroup. These connections further evoke emotions of anger, contempt and disgust, which have been linked to increased mobilisation and violence legitimation (Matsumoto & Hwang, 2012). We also found how pre‐existing grievances (e.g., those concerning immigration, child safety and political representation) were woven together through conspiracy frameworks that present coordinated elite betrayal as the root cause. These ready‐made narratives prove remarkably adaptable, capable of being applied to virtually any scenario, from the Southport attack to broader demographic change, whilst maintaining internal consistency. Conspiracy narratives here function as broad, flexible master frames that transcend specific events and can readily be applied to novel situations (Benford & Snow, 2000). Because the underlying narrative (an existential threat from a dangerous outgroup, enabled by a scheming elite) remains constant, followers are primed to interpret new events through it. Thus, new situations won't require entirely novel narratives and persuasion efforts as event‐specific details become absorbed into an existing narrative, potentially impacting the speed at which followers can be mobilised.

The multifaceted narrative dynamic challenges the traditional social psychological focus on clear ingroup–outgroup dichotomies. In our analysis, we highlighted a highly flexible narrative, where category boundaries can shift rapidly: those positioned as ingroup allies one day (such as moderate Conservatives) can become outgroup threats the next when they fail to support the desired position. This fluidity represents a significant departure from the stable category constructions typically examined in social identity research. This narrative flexibility was in part afforded by the real‐time, continuous posting afforded by the Telegram channel format, where posts reached followers as events developed, and a shorter‐form format than traditional communications between leader and followers.

### Limitations and future directions

Unlike speeches, posts broadcast on social networking sites lack a strong narrative structure with a clear beginning, middle and end. Therefore, whilst our selected time frame captured key developments during the riots, it necessarily excludes some events and posts. For example, our time frame did not include the Unite the Kingdom Rally (a ‘patriotic rally’ organised by Tommy Robinson that took place on July 27, 2024; Symonds, 2024) though references featured prominently in early posts before the escalation occurred. This limitation further highlights the interconnectedness and continuous narratives formed in these channels. We encourage future research to track how conspiracy narratives and category constructions evolve across influencers over extended periods, particularly examining how local events become incorporated into overarching frameworks like the Great Replacement theory.

In the present research, we focused on posts posted in the Tommy Robinson News Telegram channel and did not examine follower engagement with posts through shares or reactions, as data on forwards of posts was unavailable. Self‐categorisation theory emphasises the co‐construction of category boundaries through both leaders and followers (Haslam et al., 2020; Hogg, 2001); however, some platforms limit the direct engagement of audiences. There is therefore scope for future research to examine how posts from leaders are spread and engaged with in largely one‐to‐many communication styles.

Additionally, previous research identified that far‐right networks on Telegram form decentralised communities where no one leader dominates discussion, but rather sub‐communities emerge that fulfil different roles (Baele et al., 2023; Rothut et al., 2024). Future research could examine multiple influencers across platforms to understand how different actors fulfil distinct roles within decentralised networks. Research indicates varied leadership types emerge in these systems (Rothut et al., 2024), from ideological theorists to tactical coordinators to emotional amplifiers.

Furthermore, our findings support models of stochastic terrorism, suggesting that indirect mobilisation may reduce liability for the person making the speech (see also Amman & Meloy, 2024). As most of the content can be classed as “lawful but awful”, this may pose difficulties for content moderation efforts, as well as prosecuting group leaders. We encourage further research on the use of indirect mobilisation by group leaders across different case contexts and its implications for policy and practice.

### Conclusion

This research makes a timely contribution to understanding how collective action can be mobilised and violence can be legitimatised by online influencers. Through a case study of the *Tommy Robinson News* Telegram channel during the August 2024 UK riots, we extended the social identity approach to mobilisation beyond its current focus on offline settings and elected leaders (Haslam et al., 2023; Ntontis et al., 2024; but see Maxwell et al., 2025), demonstrating how platform affordances such as broadcast‐style communications alter the dynamics of group identity and norm construction. We highlight how indirect mobilisation strategies communicated through discursive identity work can legitimate violence while maintaining plausible deniability and reducing exposure to legal risk, which carries implications for legal frameworks and content moderation approaches (Amman & Meloy, 2024). As information increasingly spreads by individual voices, this research provides a novel inquiry into how identity and group norms are constructed, and how action is mobilised through one‐to‐many channel communications.

## AUTHOR CONTRIBUTIONS

**Darja Wischerath:** Conceptualization; data curation; methodology; formal analysis; writing – review and editing; writing – original draft; visualization. **Olivia Brown:** Conceptualization; methodology; writing – original draft; writing – review and editing; supervision. **Lukasz Piwek:** Conceptualization; data curation; methodology; writing – review and editing; supervision. **Brittany I. Davidson:** Supervision; funding acquisition; writing – review and editing.

## CONFLICT OF INTEREST STATEMENT

The authors have no conflict of interest for the work.

## Data Availability

The data that support the findings of this study are available on request from the corresponding author. The data are not publicly available due to ethical restrictions.

## APPENDIX A

*Most common Emoji reactions in Tommy Robinson News channel*.

**Table:**

Emoji reactions in most messages	Emoji reactions with most uses
221 messages	54,632 uses
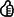
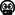
141 messages	22,848
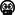
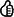
138 messages	8553
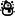
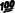
118 messages	6768
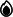
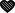
117 messages	6239
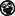
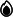
111 messages	3481
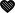
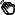
108 messages	2817
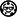
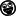
95 messages	2677
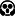
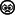
93 messages	2375
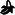
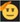
93 messages	1964
